# Video and acceleration records of streaked shearwaters allows detection of two foraging behaviours associated with large marine predators

**DOI:** 10.1371/journal.pone.0254454

**Published:** 2021-07-16

**Authors:** Aran Garrod, Sei Yamamoto, Kentaro Q. Sakamoto, Katsufumi Sato

**Affiliations:** 1 Department of Natural Environmental Studies, Graduate School of Frontier Sciences, University of Tokyo, Tokyo, Japan; 2 Department of Aquatic Bioscience, Graduate School of Agricultural and Life Sciences, The University of Tokyo, Tokyo, Japan; 3 Department of Marine Bioscience, Atmosphere and Ocean Research Institute, The University of Tokyo, Kashiwa, Japan; MARE – Marine and Environmental Sciences Centre, PORTUGAL

## Abstract

The study of seabird behaviour has largely relied on animal-borne tags to gather information, requiring interpretation to estimate at-sea behaviours. Details of shallow-diving birds’ foraging are less known than deep-diving species due to difficulty in identifying shallow dives from biologging devices. Development of smaller video loggers allow a direct view of these birds’ behaviours, at the cost of short battery capacity. However, recordings from video loggers combined with relatively low power usage accelerometers give a means to develop a reliable foraging detection method. Combined video and acceleration loggers were attached to streaked shearwaters in Funakoshi-Ohshima Island (39°24’N,141°59’E) during the breeding season in 2018. Video recordings were classified into behavioural categories (rest, transit, and foraging) and a detection method was generated from the acceleration signals. Two foraging behaviours, surface seizing and foraging dives, are reported with video recordings. Surface seizing was comprised of successive take-offs and landings (mean duration 0.6 and 1.5s, respectively), while foraging dives were shallow subsurface dives (3.2s mean duration) from the air and water surface. Birds were observed foraging close to marine predators, including dolphins and large fish. Results of the behaviour detection method were validated against video recordings, with mean true and false positive rates of 90% and 0%, 79% and 5%, and 66% and <1%, for flight, surface seizing, and foraging dives, respectively. The detection method was applied to longer duration acceleration and GPS datasets collected during the 2018 and 2019 breeding seasons. Foraging trips lasted between 1 − 8 days, with birds performing on average 16 surface seizing events and 43 foraging dives per day, comprising <1% of daily activity, while transit and rest took up 55 and 40%, respectively. This foraging detection method can address the difficulties of recording shallow-diving foraging behaviour and provides a means to measure activity budgets across shallow diving seabird species.

## Introduction

Identifying and understanding foraging behaviour in animals details how and where they find prey. This information can be used to highlight areas of ecological importance, thereby informing conservation efforts [1, 2], indicate foraging strategies and prey species [3, 4], and show the relationship animals have with their environments [5, 6]. Detecting such behaviour can be logistically difficult through direct observation, however, animal-borne biologging devices provide a means to record animal movements in their natural habitat [7]. Biologging tags recording a variety of datatypes such as pressure (depth), GPS, and acceleration, have been applied to a wide range of species. All these data require interpreting to understand the behaviours involved to make ecological inferences.

In seabird species, biologging data used to detect foraging have previously focussed on depth recordings deciphered from pressure sensor and acceleration data [8–10]. Birds diving to suitable depths provide a record of dive behaviour by recording the pressures the animal experiences. This method becomes less suitable for shallow dives as diving to shallower depths means the animals experience reduced pressure differences in short periods that are less detectable by sensors [11]. However, acceleration signals are high frequency records of motion and contain details of the tagged animal’s behaviours. The signals can be used to decipher behaviours but require a detection method, which in turn requires some validation. Previously, studies using acceleration alone to identify foraging have used automated methods [12, 13] or concurrently recorded data (depth) [11] as an indication of foraging behaviour to produce a suitable behaviour detection method. Sur et al. [14] combined acceleration, GPS, and external video recordings to classify behaviours from acceleration recordings. Use of the methods employed by these studies can be limited in specific circumstances. Short and shallow dive behaviours reduce the effectiveness of automated methods and concurrent pressure sensor data. Automated clustering of acceleration signals requires distinction in frequencies of accelerations to separate behaviours, for example using different rates of flapping to distinguish flight and take-off. However, behaviours lasting short durations can be missed using this method as the small acceleration signal samples reduce the ability to accurately identify behaviour frequencies. Similarly, shallow dives can be missed through lack of resolution of pressure sensors. Video recordings are an ideal method to identify behaviours though recording seabirds is logistically difficult given their wide-ranging nature and the larger mass of video recording tags means many bird species are too small to carry tags with sufficient battery capacity.

Continual advances in technology reduce size and mass of tag components, allowing application of tags to lighter and smaller species, including seabirds, without significantly affecting their locomotive or foraging abilities. Developments in animal-borne video camera loggers produced tags capable of visually recording bird behaviours while concurrently recording three dimensional acceleration characteristics. Thus, seabird activities can be directly observed alongside an acceleration record of body movements. This provides a means to generate and validate a behaviour detection method using video recordings.

Shearwaters are seabirds in the order Procellariiformes that are globally distributed, and exhibit a similarly wide range of foraging behaviours. Shearwater species are known to dive in order to forage, however, the characteristics of foraging dives varies considerably across the *Puffinus*, *Procellaria*, and *Calonectris* genera, with *Calonectris* species typically the shallowest divers [15]. Prior studies examining shearwater foraging in detail have described surface foraging in Scopoli’s shearwaters (*Calonectris diomedea*) [11], streaked shearwaters [4], and short-tailed shearwaters (*Puffinus tenuirostris*) [13]. Streaked shearwaters perform exceptionally short and shallow dives. Thus far, information about the foraging habits of these animals has been collated using GPS [16], and acceleration and depth data [4]. However, development of new smaller and lighter video tags can provide a new perspective on a species for whom this method was previously impractical. This study examines video footage collected by animal-borne tags alongside concurrent high-resolution acceleration data to generate a detection method to identify foraging behaviour. This detection method is also applied to longer term acceleration and GPS data to evaluate foraging characteristics of these animals.

## Materials and methods

### Field experiments

Field experiments were conducted under permission from the Ministry of the Environment and the Agency for Cultural Affairs, government of Japan, and the Ethics Committee of the University of Tokyo. Experiments were carried out on breeding streaked shearwaters (*Calonectris leucomelas*, mean body mass ± standard deviation, 560 ± 52 g, n = 25) at Funakoshi-Ohshima Island (39°24’N,141°59’E), Japan, during the chick-rearing periods of August-September 2018 and August 2019. A total of 27 birds were captured by hand at their burrows and one tag was attached to each bird. Five birds were tagged with combined video and acceleration (DVL) tags (DVL400–3DGT, Little Leonardo, Tokyo, Japan) in 2018, attached to the chest feathers using waterproof tape (Tesa, Hamburg, Germany) and instant glue (Loctite, Düsseldorf, Germany). DVL tags were attached to the chest to better observe foraging behaviours and subsurface prey or predators during landings. These loggers were used to derive the behaviour detection algorithm. Twelve other individuals were tagged with GPS and acceleration (AxyTrek) tags (Axy-Trek Marine, Technosmart, Guidonia Montecelio, Italy) to the back feathers in 2018 and 10 in 2019. AxyTrek tags recorded for considerably longer durations than the DVL tags, and so the developed behaviour detection algorithm was applied to the AxyTrek data and details of foraging behaviours were examined. Five DVL tags (1 male, 4 female) and 11 AxyTrek tags (7 male, 4 female) were successfully recovered in 2018. Nine AxyTrek tags (7 male, 2 female) were successfully recovered in 2019.

**Fig 1:**
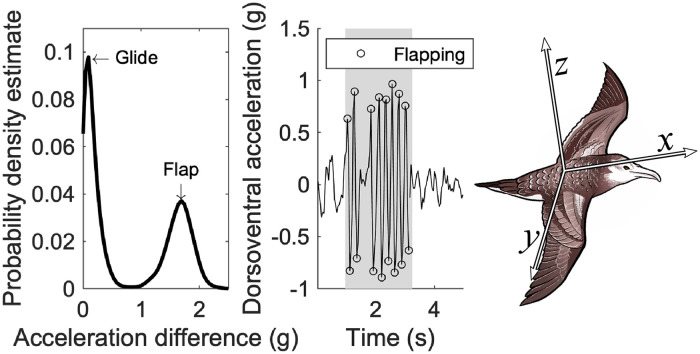
Flapping detection. An example of the flapping detection method for one AxyTrek tagged individual. The example bird (right) has three arrows showing the acceleration signals recorded and their orientation. *x* represents the longitudinal axis, *z* the dorsoventral, and *y* the lateral. The probability density estimate calculated by a kernel smoothing function of the difference in dynamic dorsoventral acceleration during estimated flight periods (left) showing flapping (smaller peak) and gliding (larger peak). Removing peaks and troughs with a difference less than the inter-peak trough isolates flapping behaviour (middle). Selected peaks and troughs (circled) separated by a duration of less than 0.5 seconds are grouped into flapping bouts (grey region).

DVL tags were set to record acceleration in three axes (longitudinal *x*, dorsoventral *z*, and lateral *y*, Fig 1) at a sample rate of 20 Hz and recorded 2 hours of continuous video at 30 fps. Acceleration was recorded from the moment the tags were attached while video recordings were programmed to begin at 12:00, 10:00, 12:00, 12:00, and 11:00 of the attachment day for each DVL tag, respectively. AxyTrek tags were set to record acceleration in three axes at 25 Hz. Ten AxyTrek tags in 2019 and two in 2018 recorded a positional fix every 5 seconds, while the other 10 AxyTrek tags in 2018 recorded a fix every 30 seconds. AxyTrek and DVL tags weigh 20g and 25g in air, respectively, <5% of the bird’s body mass.

### Video analysis

Behaviours performed by tagged birds, and their start/end times, were determined from DVL video recordings. Flight (made up of flapping and gliding) and non-flight behaviours were identified by submergence of the video camera. Two types of foraging behaviour were observed, surface seizing and foraging dives. Surface seizing was characterised by frequent take-offs and landings, during which the bird could occasionally be seen submerging its head. Take-offs during surface seizing were separated from take-offs preceding flight by whether the bird performed another landing or a glide. Foraging dives were distinguishable during video recordings by clear descent and ascent phases. The video cameras were occasionally obscured, making behaviours unable to be deciphered and birds also occasionally pecked tags during flight. Data from these periods (ranging from 19 − 28%) were not included in further analysis. Videos were viewed using VLC (VideoLAN).

### Behaviour acceleration characteristics

The behaviour detection method was derived from acceleration signals of DVL tags. Through visual inspection of the DVL acceleration recordings, a step-by-step process was generated to identify flight, rest, take-off, surface seizing, and foraging dives. This detection method was validated against the video footage.

Acceleration is comprised of static (associated to posture) and dynamic (primarily caused by propulsion from the animal) components. Static acceleration (*x*_*S*_, *z*_*S*_, and *y*_*S*_) was estimated by passing the raw signal through a low-pass filter at 1.5 Hz (filter order 100). Dynamic acceleration (*x*_*D*_, *z*_*D*_, and *y*_*D*_) was calculated by subtracting static acceleration from the raw acceleration signals. Static and dynamic components were used to categorise behaviours.

#### Identifying flight

Streaked shearwater flight is made up of flapping and glides. Flapping motion is recorded in the dynamic dorsoventral acceleration (*z*_*D*_), which contains the oscillating motion the bird experiences during wingstrokes. These oscillations are estimated by the differences in local maxima and minima of the *z*_*D*_ signal (Fig 1). This motion produces larger displacements in *z*_*D*_ than glides, which should produce little dynamic movement. Differences between local maxima and minima of *z*_*D*_ show a strong bi-modal distribution, and the inter-peak trough of this distribution can then be used to isolate flapping behaviour (Fig 1). The inter-peak trough of the differences in *z*_*D*_ maxima/minima would be the threshold to identify flaps. However, though the behaviour detection method was generated DVL tag data, we intended to apply the method to AxyTrek data. As DVL- and AxyTrek-bearing individuals were either tagged using chest- or back-mountings, respectively, the recorded acceleration signals would differ in their characteristics. Therefore, the same threshold values would not be suitable across tags and so we generated threshold values for each individual tag by estimating periods of flight from their acceleration recordings to act as reference periods to generate the thresholds.

Flight can be estimated by using *a priori* knowledge of flapping frequencies. A spectrogram of dorsoventral acceleration was generated using a Fast Fourier Transform with a 4 second window and 85% overlap. Streaked shearwaters typically flap at rates around 4 Hz during flight [17], so spectrograms should show higher energy densities around 4 Hz than for higher frequencies during flight. To estimate periods of time with relatively high energy densities at the frequencies expected during flight, we summed energy densities within two bands, the flapping flight band (3.5 to 5 Hz), and the take-off band (5+ Hz), and calculated their difference. The flapping flight band frequency range should contain the 4 Hz flapping signals typically performed during flight, while the 5+ Hz take-off band should contain higher rate flapping. Differences in energy densities between the flapping flight and take-off bands were summed in one minute moving windows. Minutes with the greatest positive difference in energy densities (where the flapping flight band energy was greater than the take-off band energy) were selected as predicted flight minutes (PFMs). Selected minutes were required to be at least 5 minutes apart to avoid selecting PFMs from the same short period of flight, generating threshold values from a wider range, and so a better encapsulation, of acceleration signals during flight. Ten PFMs were selected for each DVL tag recording and 20 for each day of AxyTrek tag recordings. Analysis was performed using custom scripts in MATLAB [18].

For each tag, differences in *z*_*D*_ maxima/minima in all PFMs were calculated and the median of the inter-peak troughs was set as the threshold to identify flaps. These flaps are then grouped into flapping bouts with no gaps less than 0.5 seconds. These flapping bouts are then further grouped to contain all flapping behaviour with no more than 30 seconds between them. Fig 1 shows an example of the bi-modal distribution in displacements between flaps and glides, and the resulting selected flapping bout.

#### Identifying take-off

During take-offs and dives/landings, birds undergo large rotations in the longitudinal axis as they align themselves upward to gain altitude or downward to reach the water surface, respectively. During flight, these rotations are likely smaller as the bird maintains a steady course. These rotations are recorded in pitch angles, which were calculated using the equation *Pitch* = arcsin *x*_*S*_ derived from a method using acceleration to identify body angle in diving seals [19]. To identify take-offs, transitions to periods of flapping are tested for presence of a large upward pitch change. Large pitch changes were estimated as 1.5 × the median of maximum differences in local pitch maxima and minima during PFMs. The large pitch change threshold for DVL tags was taken as the median of all DVL large pitch change thresholds to account for the relatively short duration (2 hours per tag) of DVL acceleration data.

#### Identifying foraging behaviours

To identify foraging behaviours, large pitch changes were also used. Large pitch changes within 23.3 seconds (the mean foraging bout duration recorded on video) were grouped. Each of these large pitch change groups were then checked for foraging dives or surface seizing. Foraging dives required a pitch angle under the median of minimum PFM pitch angles −30 degrees (representing the downward orientation), followed by a pitch angle exceeding the median of mean PFM pitches + twice the median PFM pitch variance (representing a return to the surface) within 10 seconds. Surface seizing required 3+ large pitch changes to occur within 2 seconds of one another. All remaining periods were classed as unknown. When applying the detection algorithm to AxyTrek data, upward pitch changes originating above the median of minimum PFM pitch values were removed.

#### Identifying rest

As no rest behaviour was observed during the video recordings, it was estimated from overall dynamic body acceleration (ODBA), the sum of the absolute dynamic acceleration in each axis. ODBA is commonly used as a representation of relative movement [20, 21]. A 10 second moving average of ODBA (*ODmn*) was calculated, and from visual inspection of a timeseries of *ODmn* and travel speeds of AxyTrek data 0.2g (g = 9.8*ms*^−2^) was suggested to be a suitable threshold under which rest behaviour was assigned.

#### Validation of accelerometry behaviour detection with video recordings

The detection method was performed on each DVL tags’ acceleration data. The detected behaviours were then validated by comparison to the behaviours observed on the concurrent video recordings. Validation rates (true and false positive rates) were calculated for each tag. A behaviour ethogram from video recordings sampled at the same rate as the acceleration data (behaviours assigned every 0.05 seconds) was generated. True and false positive rates (TPR and FPR, respectively) were calculated using Eq 1.

$$\begin{array}{c} \begin{array}{c} TPR=\frac{PF_{c}}{V_{F}}\\ FPR=\frac{PF_{i}}{V_{O}} \end{array} \end{array}$$ (1)

where *PF*_*c*_ is the total duration of the correctly identified behaviour, *PF*_*i*_ is the total duration of the incorrectly labelled behaviour, *V*_*F*_ is the total duration of the video-recorded behaviour, and *V*_*O*_ is the total duration of other video recorded behaviours.

### Application of behaviour detection algorithm to long duration acceleration

The behaviour detection method was applied to the long-term AxyTrek tag datasets, identifying flight, rest, take-off, surface seizing, and foraging dives. Behaviours were also grouped into three functional categories: resting, transit (flight and take-offs), and foraging (surface seizing and foraging dives). Analysis was performed on individual days of data for computing ease. Data collected while the birds were within 1.5 km from the nest colony were removed. Surface seizing or foraging dives were assigned to each GPS fix within 30 seconds of the behaviour. Speeds were calculated from Euclidean distances between GPS fixes. Due to inaccuracies in GPS locations, speeds were calculated between fixes using a 5-fix moving window. GPS fixes showing unrealistic speeds (>80 kph) were removed. Trip durations were recorded from visual observation of the data, and foraging trips were assigned as long (>2 days) or short (≤2 days). GPS fixes assigned with foraging behaviours with speeds greater than 15 kph [22] were reclassified as flight. Flight and unknown behaviours lasting less than 5 seconds were removed. Utilisation distributions of foraging behaviours and male/female foraging spots were generated for each year from grouped data of all individuals, ad hoc smoothing parameter, and 1000 × 1000 grid (approximately 400 × 500 m grid cells) with the kernelUD function (R package, *adehabitatHR*). Linear mixed effects models were used to test for differences in distance travelled or time spent foraging across sexes, and if total daily durations of behaviour categories (foraging, transit, or rest) differed between long (>2 days) or short (≤2 days) foraging trips. Individual and trip number were included as random effects. GPS data was analysed using the R statistical language [23].

## Results

### Video-recorded behaviours and presence of other predators

**Fig 2:**
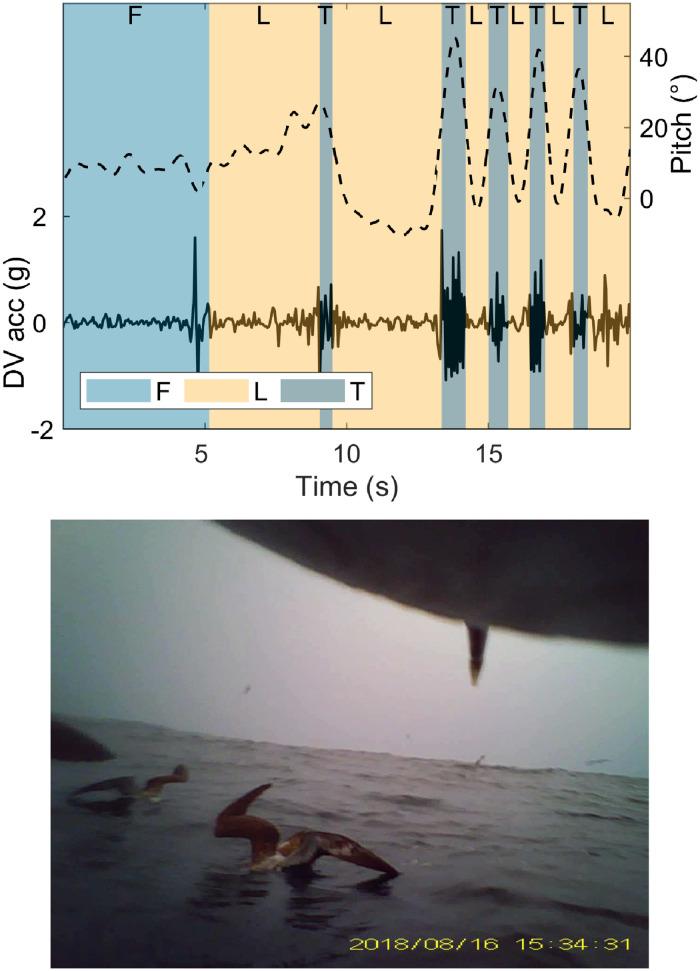
Surface seizing example. Dynamic dorsoventral acceleration (DV acc; solid line) and pitch (dashed line) during a transition from flight to surface seizing event, as recorded by video (top). The background is colour-coded depending on behaviour, flight (F), landing (L), and take-off (T), and each behaviour is labelled above. This foraging consisted of a series of short landings during which the tag was submerged separated by even shorter take-offs. The video-recordings captured some non-tagged birds performing foraging behaviours (bottom). In this example, a non-tagged conspecific clearly has its head submerged while sat on the water surface.

**Fig 3:**
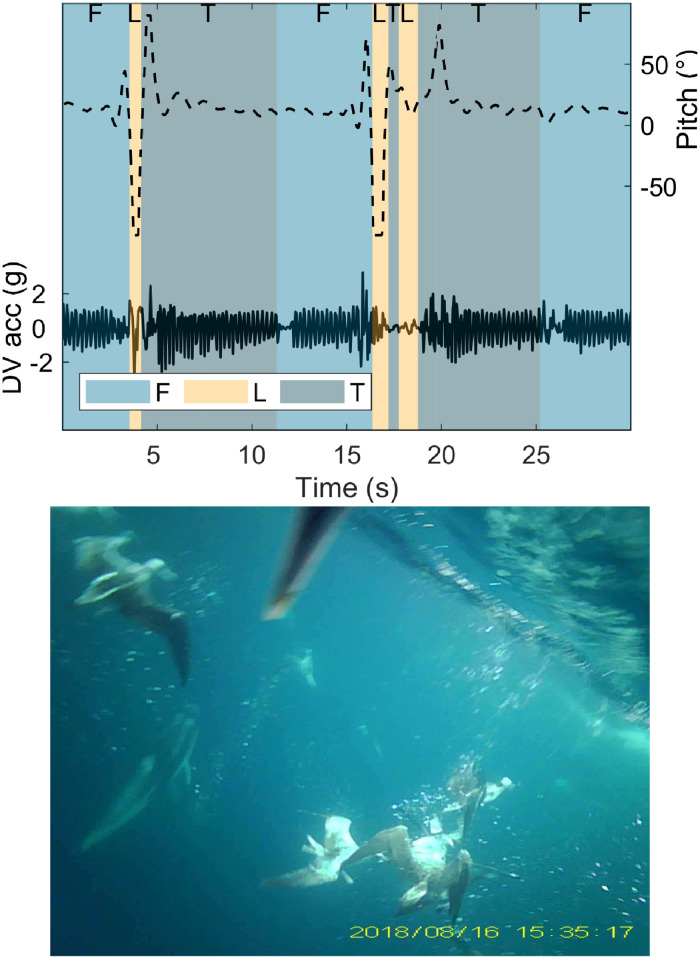
Foraging dive example. The dynamic dorsoventral acceleration (DV acc; solid line) and pitch (dashed line) during two foraging dives between periods of flight (top). The colour of the background refers to the behaviour observed, flight (F), landing (L), and take-off (T), and each behaviour is labelled above. During these landings, the water surface can be observed from underneath during the video recording, suggesting full submersion of the bird. Below is a screenshot from a video-recording showing another shearwater completely submerged during a foraging dive.

**Fig 4:**
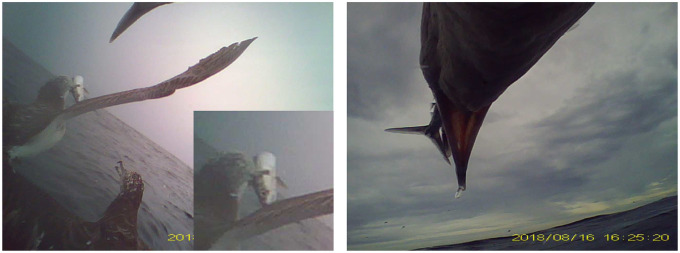
Screenshots of prey captures. A number of prey captures were observed in the DVL recordings. These examples show an unknown species capture by a conspecific which is then competed for by other shearwaters following take-off (left), and a Pacific saury caught by the tagged individual during a surface seizing event (right). This footage suggests that shearwaters capture prey then feed whilst in flight.

During video recordings, birds flew for 75.6 minutes on average (±36 standard deviation), spent 1.1 minutes taking off (±0.9), surface seized for 10 minutes (±7), performed foraging dives for 0.4 minutes (±0.7) out of the total 2 hour video durations. Video recordings contained two types of foraging behaviour: surface seizing, and foraging dives. Surface seizing consisted of landings and take-offs occurring in quick succession (Fig 2). During foraging dives the water surface was visible on the video footage as the birds ascended, indicating the birds fully submerging (Fig 3). Recordings of foraging behaviours in conspecifics showed that during surface seizing the shearwaters would submerge their heads under the water surface, as visible both from the tagged animal and in a recorded conspecific (Fig 2). DVL-tagged individuals all performed surface seizing (mean landing duration ± standard deviation 1.5±1.4s, mean take-off duration 0.6±1.6s, mean total duration including inter-landing take-offs 23.1±40.4s). Foraging dives following a plunge from the air and after an initial landing were both recorded. Four DVL-tagged individuals performed foraging dives (mean dive duration 3.2±1.2s). Video footage of both foraging behaviours can be viewed in the S1–S4 Videos). Pre-flight take-offs were considerably longer than those of surface seizing, lasting 3.0±1.3s mean duration. During the recordings, a number of prey captures, both by the tagged individual and others, were observed (two examples in Fig 4, video of prey capture S5 Video). The videos also showed large groups of conspecifics sitting on the water surface, and other marine predators, including common dolphinfish (*Coryphaena hippurus*) and Pacific white-sided dolphins (*Lagenorhynchus obliquidens*).

### Behaviour detection validation

Flight was correctly classified for 84 to 97% of tag durations (mean 90%) with no false positives, while surface seizing was correctly identified between 52 to 99% of observed surface seizing across all tags, with a mean of 79% (Table 1). False positive detections of surface seizing ranged from 1 to 7%. Most false positive detections of surface seizing events occurred from the detection method grouping surface seizing events separated by short flights. Foraging dives were correctly identified from 47 to 98% of observed foraging dive durations, and incorrectly detected for <1% of video-recorded foraging dives across all tags. False positive foraging dive detections were caused by misclassifying surface seizing behaviour. Four surface seizing events were incorrectly classified as foraging dives. Video-recorded foraging behaviour was observed during all detected foraging bouts. The detection method was designed to minimise type 1 error to ensure foraging detections were reliable though this did reduce the true positive detection rates. No rest behaviour was observed during DVL recordings, and so neither were any take-offs from rest.

**Table 1:** True positive and false positive rates of behaviour estimation of video-acceleration tags.

Tag	Flight		Surface seizing		Foraging dive	
	TPR (%)	FPR (%)	TPR (%)	FPR (%)	TPR (%)	FPR (%)
**17008**	91	0	80	6	61	< 1
**18012**	85	0	52	6	47	< 1
**18014**	84	0	68	7	98	< 1
**18017**	93	0	95	6	57	< 1
**18018**	97	0	99	1	NA	NA
**Mean**	90	0	79	5	66	< 1

Validation rates (true positive rate, TPR; false positive rate FPR) of estimated behaviours across all video and acceleration tags.

### Foraging trips

**Fig 5:**
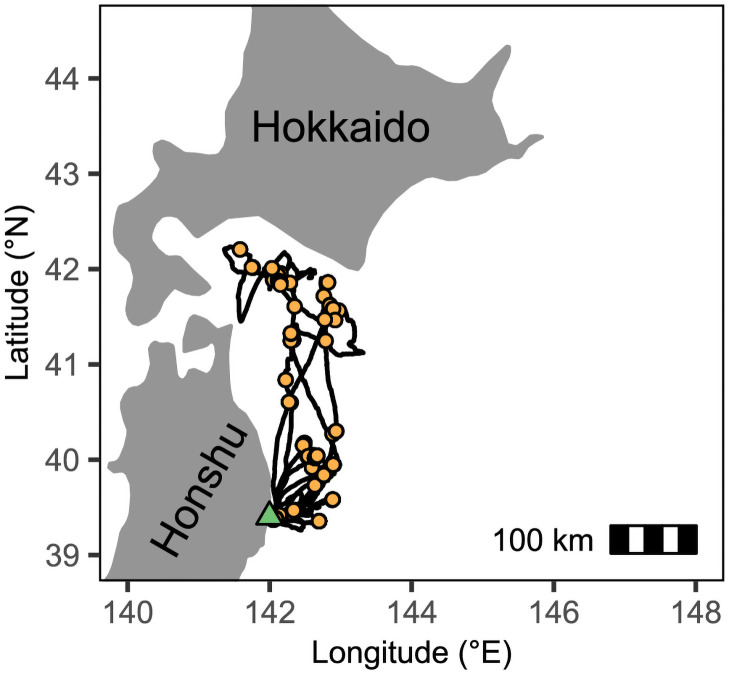
Example track and foraging locations of single individual. GPS track and foraging points (orange dots) of a single bird tracked over 12 days, 7 foraging trips. The nesting site is indicated by a green triangle.

**Fig 6:**
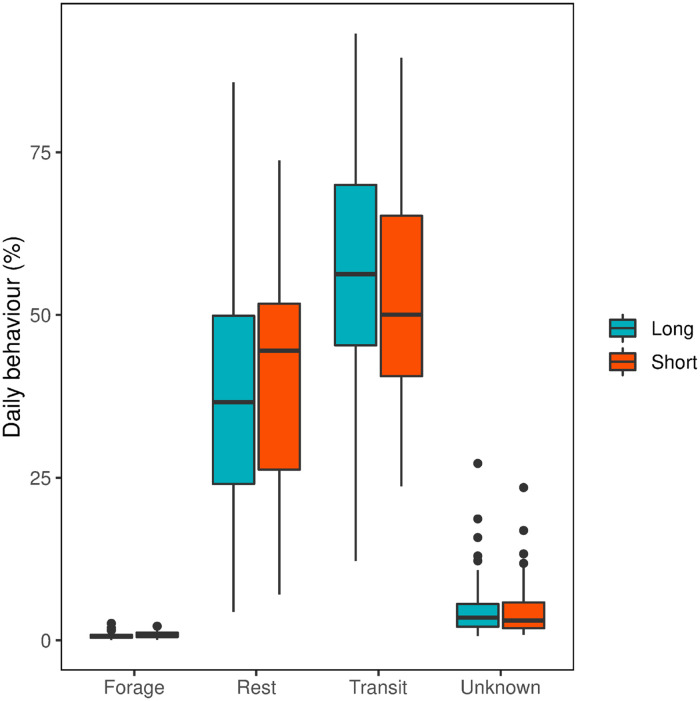
Daily proportions of behaviours detected by foraging algorithm. Proportions of 24 hour period the foraging algorithm assigned to each behaviour classification: forage, rest, transit, and unknown. Behaviour proportions are split into long (2+ days) and short (<2 days) trips. Data are presented as the median with the surrounding box edges being the 25th and 75th percentiles and the whiskers 1.5 × the interquartile range.

Forty foraging trips were made over 99 total days of recording, lasting from 1 to 8 days (example track in Fig 5). Individuals and their foraging trip durations are listed in full in Table 2. Single day trips were the most common and were recorded in all but one individual. Shearwaters performed an average 39 foraging dives per day lasting a total of 72 seconds and 53 dives lasting 102 total seconds in 2018 and 2019, respectively. Surface seizing events occurred 15 and 18 times per day lasting 481 and 448 seconds in total during 2018 and 2019, respectively. Daily activity budgets of foraging trips (Fig 6) showed that birds spent most their time in transit (median 56% on long trips, 50% on short trips), followed by resting (37% on long trips, 45% on short trips). Foraging took up 0.6 and 0.8% of long and short trips, respectively. Over 90% of foraging detections occurred at speeds (calculated via a 5-minute moving window) less than 15kph. GPS tracks and detected behaviours can be found in the Dryad Digital Repository [24].

**Table 2:** Tag deployment and foraging trip durations.

Tag	Deployment duration (days)	Trip duration (days)						
**2018**								
**2017–9-S1**	5	1	4					
**1-S2**	11	2	4	1	4			
**3-S2**	12	3	1	4	1	1	1	1
**4-S1**	10	3	1	6				
**5-S1**	9	8	1					
**6-S1**	9	4	5					
**7-S1**	11	2	5	1	1	2		
**8-S1**	10	3	1	5	1			
**9-S1**	9	2	1	4	2			
**10-S1**	6	1	4	1				
**11-S1**	6	1	3	1	1			
**2019**								
**1-S1**	4	1	1	1	1+			
**2-S1**	4	1	3					
**2018–01-S1**	5	1	1	1	1	1+		
**2018–03-S1**	5	1	4					
**2018–04-S1**	5	1	2	1	NA			
**2018–05-S1**	5	1	4					
**3-S1**	5	1	1	1	1	1+		
**4-S1**	4	1	1	1	2			
**5-S1**	5	1	1	3				

Total duration of tag deployments from the start of the first foraging trip to the end of the last recorded in 2018 and 2019. Individual foraging trip durations in days are listed. Foraging trips that stopped recording prior to the bird returning to the nest site by the end of the day are labelled with a “+” to indicate that the bird likely remained at sea for at least another day. Tag 2018–04-S1 ended recording in the morning of the fifth day, and so the foraging trip duration is labelled NA.

**Fig 7:**
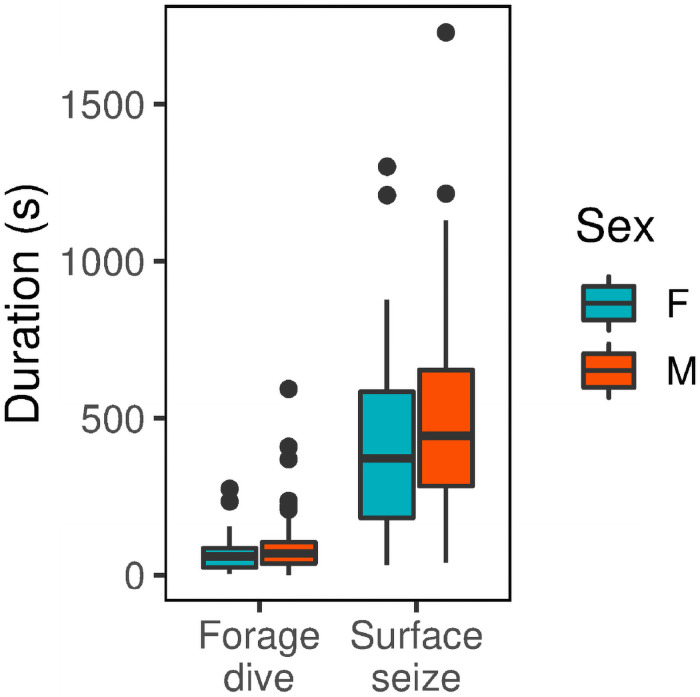
Daily foraging durations of males and females. Daily durations of diving (D) or surface seizing (S) between males and females. Males tended to forage for longer than females, however, this difference was not significant (*p* > 0.05, linear mixed model with ID and trip number as mixed effects).

**Fig 8:**
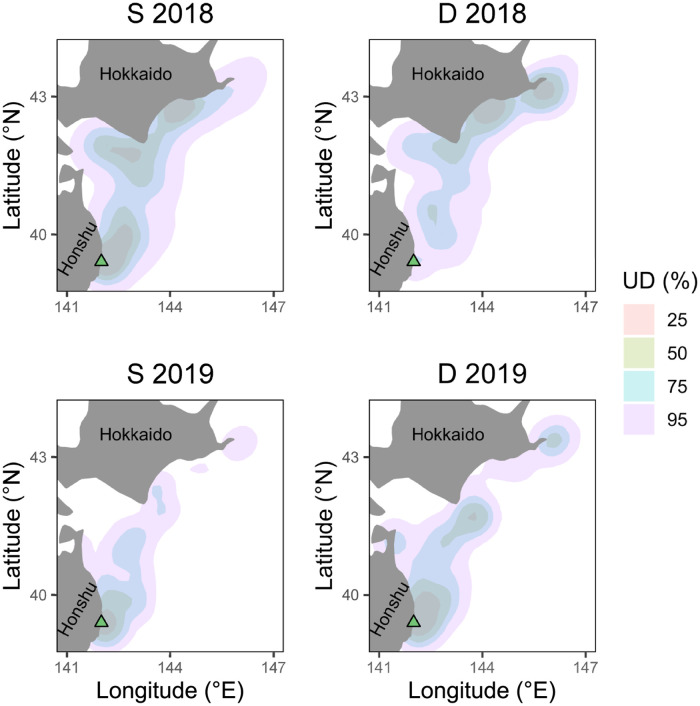
Utilisation distributions of foraging locations by behaviour. Distribution of GPS fixes with a foraging behaviour occurring within 30 seconds. Distributions are split into dives (D) and surface seizing (S). Rows are split by year, 2018 on the top, 2019 on the bottom. The nesting site is indicated by a green triangle.

On average, males travelled a greater maximum distance from the nest colony (466 km±85, mean ± sd, *n* = 7 in 2018, 321 km±160, *n* = 7 in 2019) than females (385 km±150, *n* = 4 in 2018, 163 km±75, *n* = 2) though the trip reaching the furthest distance from the nest colony (610 km) was performed by a female. Distributions of foraging behaviours differed little (Fig 8), with overlaps between utilisation distributions of foraging dives and surface seizes. In 2018, the proportion of foraging dive utilisation distributions within surface seizing distributions were 97, 83, 59, and 56% at 95, 75, 50, and 25% contours, respectively. In 2019, 70, 57, 42, and 36% of foraging dive utilisation distributions overlapped with those of surface seizing at 95, 75, 50, and 25% contours, respectively. Distance travelled from the nest colony did not differ between sexes (*p* > 0.05) while males and females did not differ significantly in daily durations of surface seizing or foraging dives (*p* > 0.05, Fig 7). Daily durations of either surface seizing or foraging dives did not differ significantly between short or long foraging trips (*p* > 0.05). Percentage of foraging trips spent foraging did not differ significantly (*p* > 0.05), however, birds flew more and rested less during long trip days (*p* < 0.05).

Behaviours that occurred most during the 5 minutes before dives were transit (66%), followed by unknown (16%), rest (14%), and surface seizing (5%), and prior to surface seizing, transit occurred most (56%), followed by rest (27%), and unknown (10%). Seven percent of surface seizing events occurred after preceding surface seizing separated by short changes in behaviour (short flights/rests between surface seizing events). The most detected behaviour in the 5 minutes following dives and surface seizing was also transit (51 and 68%, respectively). Surface seizing was the next most common behaviour after foraging dives (22%), followed by unknown (21%) and rest (7%). Unknown (9%) and rest (8%) were the next most common behaviours following surface seizing. The remaining 15% of surface seizing events were also followed by more surface seizing separated by short flights/rests.

## Discussion

In this study, we report two types of foraging behaviour in streaked shearwaters recorded on video and generate a detection method to estimate these behaviours from acceleration signals. We present video footage directly observing both styles of foraging. Foraging behaviour of shallow-diving seabirds can be underrepresented in the scientific literature due to complexity in deriving those behaviours from on-board tag data. We provide a framework to estimate shallow-dive foraging behaviour which helps address this imbalance, and demonstrate the use of the detection method to gather information on the foraging habits of streaked shearwaters nesting in northeastern Japan.

### Shallow foraging behaviours

Our study is the first to the author’s knowledge to describe foraging behaviours of streaked shearwaters in fine detail through video recordings. We observed two distinct types of foraging, surface seizing where birds do not fully submerge themselves under the water surface, and foraging dives, where birds dive into the water from either air or from the surface. The two types of foraging behaviour we describe may be deployed in different scenarios or for the capture of different prey. Drone footage (see S4 Video) shows shearwaters near the nest colony surface seizing. During the footage, large fish, believed to be chub mackerel (*Scomber japonicus*) or blue mackerel (*Scomber australasicus*) can also be observed under the water, with birds appearing to fly between landings to maintain proximity with their prey. Surface seizing may therefore allow shearwaters to prey on near-surface moving schools of prey that are clearly visible from above the water surface.

Foraging dives usually occurred between or following surface seizing behaviours. The foraging dives were concentrated around periods when large marine predators were visible. The shearwaters may have used the visual cue of another predator to intensify foraging effort. Foraging dives may be used to forage on fish that are further from the water surface. Our video recorded four successful prey captures which came as a result of rapid landings (see S5 Video). Following prey capture, the birds ingested the fish when in flight. The short contact between the birds and the water surface when capturing prey indicates that the prey were very close to the water surface when captured.

Diving procellariiformes can employ underwater flapping to propel themselves during dives. Indeed, this behaviour has been used in dive identification in the past [13]. This propulsion allows the birds to reach or maintain sufficient depths to forage. The acceleration signals of foraging dives in this study show no clear evidence of continual subsurface flapping, only initial flapping motion as the birds enter the water (Fig 3). The short durations of foraging dives also suggest limited propulsion to reach or maintain greater depths. As dives were observed originating from both plunges from the air and diving after landing on the water surface, the birds are likely diving to very shallow depths. This agrees with prior literature showing the genus *Calonectris* to dive to significantly shallower depths than *Puffinus* shearwater species [15]. Reduced ability to reach greater depths limits prey availability as capture requires prey species to be found in the upper levels of the water column.

### Marine predator association

During video recordings, large marine predators can be seen both over (porpoising dolphins) and under the water surface. During these periods, the tagged individuals increased their foraging intensity, increasing the number of surface seizing and foraging dives, as well as foraging dive duration. Associations between seabirds and marine predators have been previously reported [25–28] and are due, in part, to the effect of marine predators on the accessability of prey. Marine predators feeding from underneath force fish to the surface. This grants seabirds greater access to prey that, particularly for shallow-diving species, they may be less capable of reaching without the upward forcing of prey by marine predators. Foraging marine predators and/or conspecifcs also act as visual cues for the presence of prey [29, 30]. During video recordings, conspecifics and marine predators were visible from the air, resting and/or foraging. These visual cues can direct the shearwaters to intensify foraging effort in that area.

### Acceleration-based behaviour detection

Detection of shallow-dive foraging behaviour has proven difficult in the past as pressure sensors do not have the resolution required to detect the small signal changes produced during short dives or landings [11]. In this study, we used acceleration data with a high resolution to estimate behaviours based on acceleration signals resulting from the tagged individuals’ movements. The use of tags that record both video and acceleration allowed for validation of detected behaviours through recorded behavioural footage. This direct observation of seabirds’ behaviours during acceleration recordings is rare in the scientific literature and only achievable due to advances in miniaturised video data loggers. Our validations show that the behaviour detection method we developed was capable of reliably categorising flight (90% TPR, 0% FPR), surface seizing (79% TPR, 5% FPR), and foraging dives (66% TPR, <1% FPR) in streaked shearwaters. Foraging behaviours detected in AxyTrek data were largely (>90%) under speed thresholds for foraging in streaked shearwaters [22]. Developing a successful behavioural detection method is important for future ecological studies regarding this or similar species. Accurately quantifying foraging behaviour is necessary to not misrepresent findings of ecological studies, and the potential to underestimate foraging behaviour in shallow-diving birds is greater when using methods previously developed for deeper diving birds. This study’s detection method provides an alternate that should reduce this risk of underestimation.

### Detection method characteristics

The low false postive rates of the detection method reflect the reliability of detected foraging behaviours. We focussed on reducing type 1 error (false positives), and therefore the detection method was strict enough to ensure a high likelihood of correctly identified foraging. Our results suggest the detection method reliably indicates foraging behaviour, and the results from analysing AxyTrek data should suitably reflect the shearwaters’ foraging spots. Although foraging may have been underestimated, it is unlikely, given the relatively high true positive rates of our study, that inclusion of all foraging behaviours would significantly change the activity budget values. The method provides understanding of how and where these birds are focussing their foraging efforts.

True positive rates of both surface seizing and foraging dives varied considerably across the DVL tags. The variability in true positive rates of surface seize detection was caused by presence of foraging dives in the midst of surface seizing bouts. The detection method initially identified the foraging dives first. The surface seizing behaviours that remained were therefore cut into shorter periods. Detection of surface seizing bouts required presence of 3+ large pitch changes within 2 seconds, so shorter surface seizing bouts were more likely to be misclassified. Similarly large variability in foraging dive detection rates were due to the low total number of observed foraging dives (min 0, max 11) which caused relative true positive rates to fluctuate greatly. Despite these fluctuations, visual inspection of the acceleration signals of AxyTrek foraging detections, as well as the speeds the birds were travelling at, indicated the detection method performed well.

Behaviour estimation using acceleration data is relatively common in biologging studies. Accelerometers are becoming more ubiquitous across tags through miniaturisation and increased efficiency of battery capacity and writing to memory. Similarly, automated methods to analyse these data have become common in the scientific literature [12, 31, 32]. Use of these unsupervised methods allows acceleration data to be easily analysed without time-consuming examination of the data by hand. However, these methods require clear distinction in acceleration signals between behaviours. Application of k-means clustering as per [12] to the DVL dataset was unsuccessful due to the extremely short duration of foraging behaviours and relative similarity in their signals to those of flight. A previous study [11] reported issues identifying foraging in shallow-diving Scopoli’s shearwaters due to pressure sensors being unable to accurately detect dives lasting <2 seconds, but were successful in their detection by using acceleration data. The method they report was also unsuccessful in detecting streaked shearwater foraging, due to the thresholds not being applicable to our data. The method identifies dives using a threshold of -1 g in the longitudinal acceleration. Our recordings rarely passed this threshold, with most surface seizing behaviour being missed, and so a custom detection method was generated.

It is worth noting that the sample size in this study was low, and also particularly skewed in sex, with considerably fewer females tagged than males, particularly in 2019. As such, the ecological findings of this study may be limited due to their low sample size and so additional data to complement that collected for this study would allow for a deeper investigation into the foraging ecology of these animals.

### Foraging trip characteristics

The disparity in trip durations across 2018 and 2019 is clearly reflected in the foraging spot concentrations, and may be due to the tagging experiments in 2019 occurring earlier in the breeding season than 2018. Streaked shearwaters, like many pelagic seabirds, perform a dual foraging strategy, using short foraging trips to provision the chick, and longer trips to self-provision [33–36]. As chicks grow through the breeding season, parents are able to perform longer foraging trips, with chicks able to withstand longer periods between feedings. This is reflected in the greater distribution of foraging behaviour further from the nest colony in 2018 and the use of near-colony foraging grounds in 2019.

The activity time budget generated by the behaviour prediction assigns a small proportion of time to foraging, with most time during foraging trips devoted to flight or rest. Our results show agreement in the proportion of daily rest behaviour with those of shearwaters from the same nest site in 2010 [16]. During longer trips, birds flew more and rested less, however, there was no change in proportion of daily foraging. This is in agreement with the overlaps between both surface seizing and foraging dives in their spatial distributions. Shearwaters foraging in the same region as those of this study showed a change in diet composition in response to length of foraging trip [4]. No significant change in daily durations of surface seizing or foraging dives between short and long trips suggests shearwaters use both behaviours to forage for a variety of prey species.

Both foraging behaviours were followed and preceded most by flight in the surrounding 5 minutes. This reflects travel to or from the foraging area, or the birds’ continued search while foraging. However, rest was the second most common behaviour prior to foraging. This suggests the shearwaters performed ‘sit-and-wait’ foraging, where birds sit on the water surface waiting for prey to become available for capture [37–39].

In all shearwaters, some foraging occurred around 04:00 close to the nest site (<10 km from the colony). Foraging at this location was typically made up of surface foraging with few dives occurring. This time and location aligns with the presence of set nets and Pacific saury fishing vessels. These vessels are equipped with lighting rigs that are visible from the nest colony and would provide a stable foraging ground for streaked shearwaters. Surface seizing near fishing vessels, where the birds capture remnants of vessel catches, can be seen in their high spatial concentration near the nest colony in both years (Fig 8).

### Effects of tag mass

Biologging studies can suffer from unintended effects of tag attachments altering behaviour of individuals. Impacts of tags vary both across taxa and individuals [40]. The effect of tags increase with mass and at 5% can have significant effects on lengths of foraging trips, though not the proportion of time spent foraging or resting [40]. Our study may therefore be subject to differences in foraging trip characteristics, however, the foraging behaviours we report are likely to reflect those performed by unencumbered individuals.

### Future steps

The detection method generated by this study would benefit from a greater pool of video recordings. The relatively short duration of the video tags reduced the number of observable behaviours, with little rest behaviour detected throughout their deployment. This study therefore makes the assumption that rest behaviour would likely be observable through reduced acceleration magnitudes. A larger dataset of video and acceleration recordings would increase the accuracy and reliability of the derived detection method. Similarly, the small sample size, both in number of individuals and duration, of longer term acceleration and GPS data obtained during this study curtails findings on trends in male/female foraging and use of foraging behaviours. However, it does provide unique paths for future studies looking specifically at foraging or search behaviour.

This study occurred during the breeding season to optimise tag retrieval. The findings we make on the foraging characteristics and distributions of these animals are therefore limited to the breeding seasons only. Development of miniature satellite-relaying loggers for seabirds would allow in-depth research into foraging behaviour during non-breeding seasons and testing of changes to foraging strategy when the birds are not limited by proximity to a nest colony, and are only self-provisioning.

With increasing tag capabilities and memory and battery capacities, combining fine-scale behaviour classifications with alternate analysis methods could provide novel insights into the foraging and energetic ecology of seabirds. Custom video and GPS tags [41] attached to streaked shearwaters, where video recordings were turned on when on-board processing estimated area-restricted search behaviour occurred, focussed the video recordings to periods related to foraging. Developing the method presented in this paper could allow a similar application of on-board processing of acceleration data to record foraging behaviour, increasing the sample size of recorded foraging behaviours in streaked shearwaters and other shallow-diving seabirds.

Implementing current knowledge from ‘hand-crafted’ algorithms could update machine learning methods for greater effectiveness for shorter duration and/or shallower dives. Understanding what acceleration characteristics are of most importance, and what patterns to search for, can expand the capabilities of supervised and unsupervised machine learning. With additions of multi-modal data, algorithms generated by hand are less feasible. Better-informed machine learning methods can provide a solution to this issue.

## Conclusion

Our study presents the first occasion of combined video and acceleration data to report details of fine-scale foraging behaviour in a shallow diving seabird. We provide a framework for the detection of two different foraging behaviours observed in our study. This detection methodology is tested on observable behaviours and validated, showing strong reliability. The precise nature of the foraging detection allows precise detail of foraging descriptions, providing a means for future studies to investigate foraging ecology and decision-making in the moments leading up to foraging efforts. These findings can be important in understanding seabird ecology, and impacts of changes to their environment on seabird populations.

## Supporting information

S1 Fig Utilisation distributions foraging locations by sex for each year. Utilisation distributions calculated at 30km bandwidth of predicted foraging behaviour. Data from 2018 are displayed in the top row, 2019 in the bottom. Distributions are split by sex (female: F, male: M). The nesting site is indicated by a green triangle.
(EPS)

S1 Video Foraging dive from air. Footage of a foraging dive following a short take-off. During the dive, Pacific white-sided dolphins (*Lagenorhynchus obliquidens*) can be seen. Prior to this dive, the dolphin could be observed surfacing, and its presence may alert the shearwater to a high probability of prey being available. During this recording, a number of foraging dives were recorded in this area, as well as a number of conspecifics diving and sitting on the water surface.
(MP4)

S2 Video Foraging dive from the surface. A foraging dive performed from the water surface. The lens is clearly under the water surface after which the shearwater performs a dive where the water surface can clearly be seen from underneath. Pacific white-sided dolphins are again visible during the dive.
(MP4)

S3 Video Surface seizing. Surface seizing behaviour, showing successive landings and take-offs. The shearwater’s bill can occasionally be seen under the water surface, suggesting the birds put their heads under the water, perhaps as prey capture attempts.
(MP4)

S4 Video Surface seizing drone footage. Drone footage of surface seizing behaviour from near the nest colony. Large fish can be seen under the water surface, pushing prey fish to the surface which allows the shearwaters to capture prey with greater ease. The birds land and take-off, following the fish school while landing.
(MP4)

S5 Video Surface seizing prey capture. Footage of a rapid surface-seized prey capture of a Pacific saury (*Cololabis saira*) which is then ingested during flight.
(MP4)
